# The Multifaceted Interface Between Cytokines and microRNAs: An Ancient Mechanism to Regulate the Good and the Bad of Inflammation

**DOI:** 10.3389/fimmu.2018.03012

**Published:** 2018-12-21

**Authors:** Silvia Garavelli, Veronica De Rosa, Paola de Candia

**Affiliations:** ^1^IRCCS MultiMedica, Milan, Italy; ^2^Laboratorio di Immunologia, Istituto di Endocrinologia e Oncologia Sperimentale, Consiglio Nazionale delle Ricerche (IEOS-CNR), Naples, Italy; ^3^Unità di NeuroImmunologia, Fondazione Santa Lucia, Rome, Italy

**Keywords:** microRNAs, cytokines, CD4^+^ Th cell subsets, inflammation, autoimmunity

## Abstract

MicroRNAs (miRNAs) are evolutionary conserved small non-coding RNA molecules that affect gene expression by binding to target messenger RNAs and play a role in biological processes like cell growth, differentiation, and death. Different CD4^+^ T cell subsets such as Th1, Th2, Th17, and T regulatory cells, exert a distinct role in effector and regulatory-type immune responses. miRNAs have been shown to respond to dynamic micro-environmental cues and regulate multiple functions of T cell subsets including their development, survival and activation. Thus, miRNA functions contribute to immune homeostasis, on the one side, and to the control of immune tolerance, on the other. Among the most important proteins whose expression is targeted by miRNAs, there are the cytokines, that act as both key upstream signals and major functional outputs, and that, in turn, can affect miRNA level. Here, we analyze what is known about the regulatory circuit of miRNAs and cytokines in CD4^+^ T lymphocytes, and how this bidirectional system is dysregulated in conditions of pathological inflammation and autoimmunity. Furthermore, we describe how different T cell subsets release distinct fingerprints of miRNAs that modify the extracellular milieu and the inter-cellular communication between immune cells at the autocrine, paracrine, and endocrine level. In conclusion, a deeper knowledge of the interplay between miRNAs and cytokines in T cells may have pivotal implications for finding novel therapeutic strategies to target inflammation and autoimmune disorders.

## Introduction

MicroRNAs (miRNAs) are small (~22 nucleotides in length), non-coding RNAs, processed from longer transcripts, the pri-miRNAs, first cut to form a stem-loop structure, the pre-miRNAs. These molecules are then further processed to form the mature miRNA duplex by the subsequent action of two type III RNA endonucleases, Drosha (nuclear), and Dicer (cytoplasmic). The miRNA duplex is loaded into the Argonaute (Ago) protein to form a mature RNA interference silencing complex (RISC). The mature single stranded miRNA pairs to sites usually within the 3′ untranslated region of messenger RNAs (mRNAs), causing mRNA decay and block of translation. A detailed description of miRNA biogenesis goes beyond the scope of the present review but can be found elsewhere (1). miRNA pathway, possibly derived from the ancient RNA interference (RNAi) pathway, is common to all eukaryotes and highly conserved. One of the first miRNAs discovered, *lethal-7* (let-7), a regulator of developmental timing in *Caenorhabditis elegans*, shows a correspondent temporal expression in bilaterian animals and is crucial in regulating mammalian developmental differentiation and glucose metabolism (2–5). In humans, almost two thousand different miRNAs are known and the majority of mRNAs are miRNA conserved targets (6). This broad regulation of the transcriptome expression potential suggests miRNAs may influence all physiological and pathological processes.

A major research effort has investigated the specific impact of miRNAs on the immune system. We will here focus on a population of T lymphocytes, CD4^+^ T helper (Th) cells, crucial in orchestrating CD8^+^ T and B cell-dependent adaptive immune response. T cell receptor (TCR) stimulation, the cytokine milieu and co-stimulatory signals together lead to naïve Th cell proliferation and differentiation into effector subtypes, characterized by specific transcription factors, cytokine fingerprints, and pathogenic targets (7). Th1 cells are defined by the master regulator T-bet, produce high levels of Interleukin (IL)-2 and interferon (IFN)-γ and direct immunity toward intracellular bacteria and viruses; Th17 cells, promoted by the expression of the master regulator Rorγt, combat extracellular bacteria, and fungal infections by releasing IL-17; the master regulator Gata3 drives the differentiation of Th2 cells, which produce IL-4, IL-5, and IL-13 and recognize extracellular parasites. Follicular helper T cells (Tfh), characterized by the activity of the master regulator Bcl-6, are located within B cell follicles of secondary lymphoid organs, mostly secrete IL-4 and IL-21 and are responsible for the maintenance of germinal centers and the development of humoral immunity. CD4^+^CD25^high^FOXP3^+^ regulatory T (Treg) cells represent a functionally distinct lineage committed to exert an anti-inflammatory/immune suppressive control and sustain immunological homeostasis (8). Treg cells act by inhibiting the action of the pro-inflammatory counterpart CD4^+^ Th1 and Th17 (also referred to as T conventional or Tconv) cell subsets by the production of IL-10, IL-35, and transforming growth factor (TGF)-β. Although the categorization of Th subpopulations is useful, the reported existence of cells with cytokine signatures and functional properties intermediate between the described subsets indicates a certain degree of plasticity (9, 10).

Since the dysregulation of cytokines is associated to deranged inflammation, effector Th cell differentiation/activation must be strictly regulated in order to avoid exaggerated and/or pathological responses (11). Beside epigenetic remodeling and lineage-restricted transcription factors, miRNA-dependent regulation is now recognized to significantly modulate Th gene expression and cytokine-related functional outputs. In this minireview, we will analyze relevant data on miRNA-based networks that regulate the tuned release of specific cytokines by Th subsets, central to mount efficacious immune responses and maintain immune homeostasis.

## Global miRNA Modulation During CD4^+^ T Cell Development and Differentiation

During T lymphocyte development, miRNA pool is highly dynamic, ranging from around 30,000 to ~5,000 copies per cell when comparing the highly proliferative CD4CD8 double negative to the double positive lymphocytes undergoing selection. The miRNAs:total RNA ratio steadily increases during maturation, suggesting that miRNA suppressive potential is also regulated in terms of quantity relative to ribosomal and messenger RNA (12). Furthermore, when Th cells are TCR-stimulated, the RNA yield per cell increases with many housekeeping mRNA transcripts being induced. In parallel, global miRNA expression significantly diminishes, even before any cell division; this down-regulation depends on both pri-miRNA transcription decrease and RISC activity decline secondary to a massive Ago ubiquitination and subsequent proteasome-dependent degradation (13).

**Figure 1:**
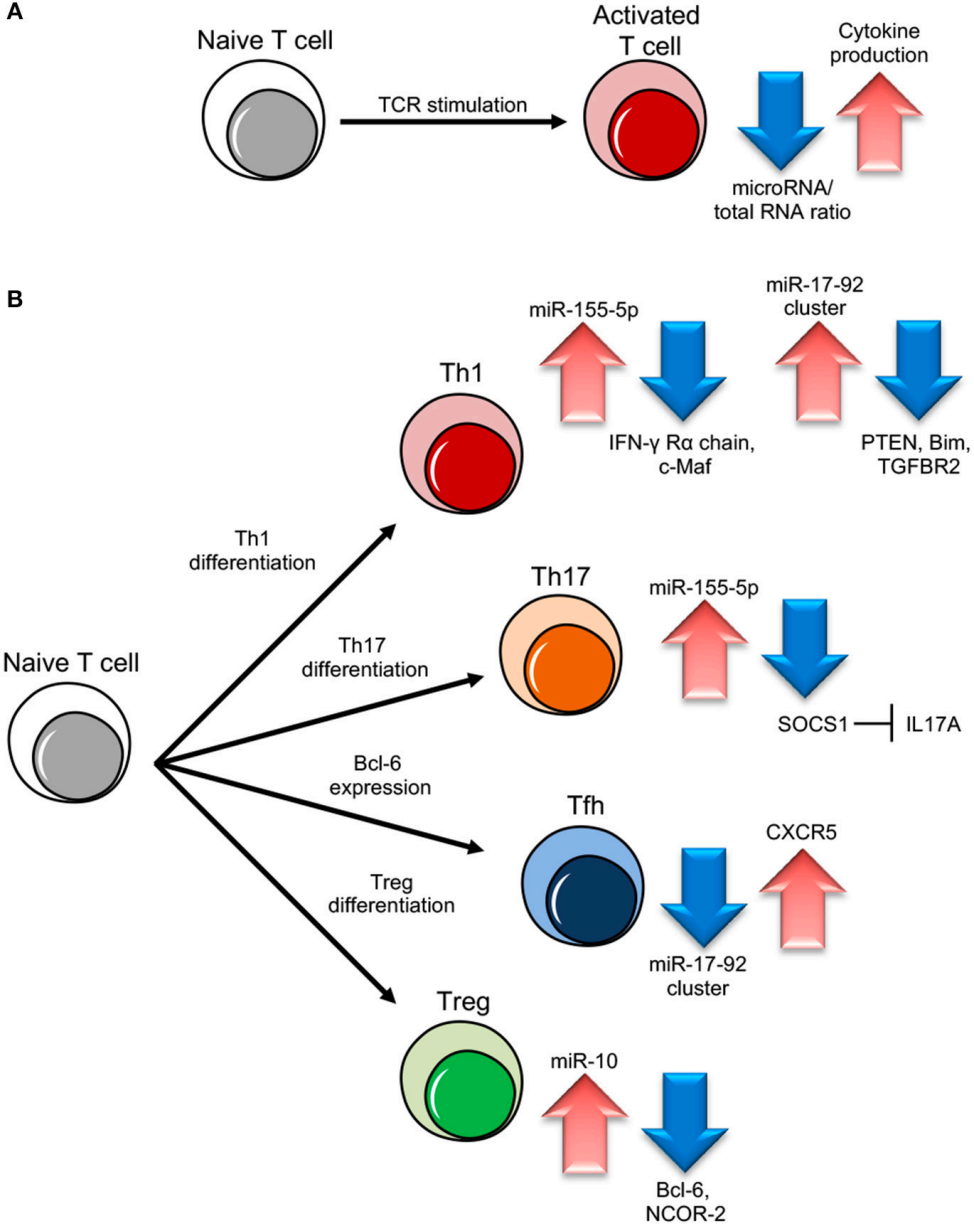
miRNA role in different T cell subset differentiation. **(A)** Upon naive T cell activation, increase in cytokine production is dependent on miRNA/total RNA ratio decrease. **(B)** During Th1 cell differentiation, up-regulation of miR-155-5p and miR-17-92 results in suppression of IFN-γ Rγ chain and c-Maf and PTEN on one side and Bim and TGFBR2 on the other, important to block Th2 differentiation and unlock cell proliferation respectively. During Th17 differentiation, miR-155-5p induction leads to SOCS1 inhibition, which in turn unleashes IL17A production. During follicular helper T cells (Tfh) cell differentiation, CXCR5 up-regulation, important for migration into follicles, is dependent on miR-17-92 cluster downregulation. During Treg cell differentiation, the increase of miR-10 expression blocks the expression of Bcl-6 (Thf differentiation) and NCOR-2 (Th17 maturation).

Ablation of the machinery for miRNA biogenesis during thymocyte differentiation or Th cell activation has devastating effects, demonstrating the critical role miRNAs play during Th gene expression reprogramming. Dicer or Drosha deletions in murine Th cells result in aberrant development, differentiation and cytokine production. Dicer deficient Th cells are not only unable to engage robust proliferation upon stimulation while actually undergoing increased apoptosis, but also show the preferential expression of IFN-γ, indicating a skewed subset commitment toward the Th1 lineage (14–17). Consistently, when miRNAs are depleted due to Ago deficiency, Th are more prone to differentiate into cytokine producing cells, suggesting that miRNA down-regulation promotes acquisition of effector functions by relaxing the repression of genes that direct Th cell differentiation, like cytokines and/or cytokine regulators (13) (Figure 1).

miRNA maturation pathway is also necessary for the development of thymic Treg cells and the induction of FOXP3 by TGF-β. Treg specific deletion of Dicer or Drosha shows a dramatic output, with the development of a lymphoproliferative phenotype resembling the one observed in the absence of FOXP3 itself (18–20).

## miRNAs on the Cusp of the Gene Expression Networks Controlling CD4^+^ Th Cell Function

The most prominent feature of Th cell differentiation is based on cytokine and transcription factor feedback loops that polarize gene expression. Th cell fate is sensitive to subtle changes of these regulatory circuits and therefore particularly responsive to miRNA regulation. Accumulating studies ablating and/or overexpressing single miRNA molecules or miRNA clusters are dissecting miRNA salient action in Th subset differentiation.

### miR-17-92 Cluster

A milestone work has been conducted on the mir-17–92 cluster, that encodes miR-17, 18a, 19a, 19b, 20a, and 92 inside a single polycistronic transcript. This cluster sustains lymphocyte proliferation and inhibits cell death by targeting the tumor suppressor phosphatase and tensin homolog (PTEN) and the proapoptotic protein Bcl-2-like protein 11, commonly named Bim; indeed, lymphocyte-specific transgenic mice over-expressing the cluster die as a consequence of lymphoproliferative disease and autoimmunity (21, 22). In particular, the mir-17–92 cluster pushes toward a more pronounced pro-inflammatory type-1 phenotype, with increased IFN-γ production and, upon viral infection, miR-17–92 expression is required for clonal expansion of virus-specific Th1 and memory formation (23, 24). Two cluster members, miR-17 and miR-19b, are the key players controlling Th1 responses, supporting IFN-γ production and suppressing inducible Treg differentiation, with PTEN and TGFβ receptor 2 (TGFBR2) as the functionally primary targets of miR-19b and miR-17, respectively (25) (Figure 1). These two miRNAs are also essential during the induction of graft-vs.-host disease (GVHD) in mice, as the systemic administration of antagomir to block either one of the two significantly inhibits alloreactive T-cell expansion and IFN-γ production, and prolongs survival (26). During Tfh cell differentiation, the master transcription factor Bcl-6 represses miR-17, miR-18a, and miR-20a and thus releases their repression on C-X-C motif chemokine receptor 5 (CXCR5), required for the migration of cells into follicles (27) (Figure 1). On the other hand, in a viral infection model, miR-17–92 acts as a critical regulator of Tfh cell differentiation by restraining the expression of genes “inappropriate” to this cell subset (28); in an airway inflammation model *in vivo*, miR-18a specifically targets three key transcription factors in the Th17 gene-expression program small mother against decapentaplegic 4 (SMAD4), hypoxia inducible factor 1α (HIF1α), and retinoid-related orphan receptor α (RORα), and blocks the differentiation of tissue Th17 cells expressing C-C chemokine receptor 6 (CCR6), RORγt, and IL-17A (29).

### miR-155

Another relevant example of a miRNA with dramatic effect on Th subset differentiation is miR-155, which maps within an exon of the non-coding RNA *bic*. This gene is found highly expressed in activated B and T cells and lymphomas and miR-155 transgenic mice develop B cell malignancies (30–33). In conditions of miR-155 deficiency, CD4^+^ Th cells proliferate normally upon TCR-stimulation but show a significant reduction of Th1 commitment and IFN-γ production and an increase in the number of IL-4 producing cells. miR-155 ability to skew Th differentiation away from the Th2 phenotype and attenuate Th2 cell responses *in vivo* depends on its capacity to directly suppress the transcription factor c-Maf, a potent trans-activator of the IL-4 promoter (34) (Figure 1). miR-155 is also able to promote Th1 differentiation and IFN-γ release through the modulation of the IFN-γ signaling by directly targeting IFN-γRα chain (Figure 1). Gain and loss-of-function analysis showed that miR-155 also positively regulates Th17 differentiation and induces the release of IL-17A through Janus kinase/signal transducer and activator of transcription (JAK/STAT). The direct target was suggested to be the suppressor of cytokine signaling 1 (SOCS1), which negatively feedbacks cytokine signal transduction (35) (Figure 1). Interestingly, in Th2 inducing conditions, miR-155 becomes unable to suppress the IFN-γRα messenger possibly because of preferential binding to high affinity Th2 specific mRNA targets, such as c-Maf, or because this suppression requires additional factors, including other miRNAs, exclusively expressed in Th1 cells (36).

## The Bidirectionality of Cytokine-miRNA Relationship

A pivotal study has described the pleiotropic effect of TGF-β on the miRNome. SMADs, signal transducers of TGF-β, promote the expression of a plethora of miRNAs by facilitating the cleavage by Drosha, through the recognition of a consensus sequence within the stem region of miRNA primary transcripts, illustrating that TGF-β gene regulation also relies on miRNA modulation (37). Another example of cytokine-dependent miRNA regulation is recordable during the switch from a resting state to clonal expansion of antigen-activated Th lymphocytes, when the suppressor of proliferation Forkhead box protein O1 (FOXO1) is initially inactivated by post-translational modifications, and then post-transcriptionally inhibited by IL-2-induced miR-182 (38).

An intriguing case of miRNA-cytokine tango is that of miR-29a and IFN-γ. A wide screen for miRNA function in primary Th cells identified miR-29 as able to correct the aberrant IFN-γ expression associated with global miRNA deficiency. This miRNA targets both T-bet and EOMES, two transcription factors known to induce IFN-γ production, but it also suppresses IFN-γ production by directly targeting its mRNA (39).

The transgenic expression of a “sponge” target to compete with endogenous miR-29 targets in *Listeria monocytogenes* infected mice increased IFN-γ serum concentrations and decreased infection burdens, further suggesting that miR-29 suppresses immune responses to intracellular pathogens by targeting IFN-γ (40). The direct involvement of miR-29 in IFN-γ regulation remains controversial, as no correlation between miR-29a and IFN-γ expression of Th cells was observed in patients during active tuberculosis in more recent works (41, 42).

## miRNA Regulation of Treg Cell Identity and the Control of Immune Homeostasis

In 2010, it was demonstrated that a single miRNA can control immune homeostasis. Treg specific deletion of miR-146a-5p resulted in a breakdown of immunological tolerance manifested in fatal IFN-γ dependent lesions in a variety of organs, associated with the augmented expression and activation of the direct target Signal transducer and activator of transcription 1 (STAT1) (43). Another study confirmed that miR-146a null mice lose peripheral T cell tolerance and die prematurely of a spontaneous autoimmune disorder, characterized by splenomegaly, lymphadenopathy, and multiorgan inflammation (44). miR-146a is part of a regulatory negative feedback loop that controls TCR signaling to NF-κB and the resolution of Th responses: mice Th cells lacking miR-146a are hyperactive in both acute antigenic and chronic inflammatory autoimmune responses because in physiological conditions TCR-driven NF-κB activation up-regulates the expression of miR-146a, which in turn down-regulates NF-κB activity, at least partly through repressing the NF-κB signaling transducers TNF receptor-associated factor 6 (TRAF6) and IL-1 receptor-associated kinase 1 (IRAK1) (45). Upon Treg induction, TGF-β is able to specifically induce miR-10a. By simultaneously targeting the transcriptional repressor Bcl-6 and the corepressor nuclear receptor corepressor 2 (NCOR2), miR-10a hampers the phenotypic conversion of Treg into Tfh cells and at the same time blocks differentiation into the Th17 subset. In other words, TGF-β can fine-tune the plasticity and fate of Th cells also through the specific induction of a single miRNA (46) (Figure 1). Notably, although under basic conditions miR-17–92-deficient Treg cells are able to maintain immune homeostasis, the expression of miR-17–92 cluster (above described as central for Th1 differentiation) reveals to be also critical for the accumulation of activated antigen-specific Treg, the differentiation into IL-10-producing effector cells and clinical remission from experimental autoimmune encephalomyelitis (EAE, a model of human multiple sclerosis) (47). Furthermore, while Treg cells do not seem to need miR-155 to exert their suppressive function, FOXP3 positively regulates miR-155 expression and this miRNA deficiency impairs Treg development by increased levels of SOCS1 and reduced responsiveness to IL-2 (48, 49).

## Genetic Basis of miRNA Regulation

A remarkable work of miRNA expression quantitative trait loci (miR-eQTL) analysis discovered that most of these loci are located upstream of their associated intergenic miRNAs by mapping more than five thousand individuals. Cis-miR-eQTLs miRNAs display differential expression in relation to the corresponding trait, and distal regulatory elements may also affect interindividual variability associated with a variety of complex traits (50). A single-miRNA based meta-analysis has extensively reviewed studies suggestive of an association between the miR-146a single nucleotide polymorphisms (SNPs) and susceptibility to autoimmune diseases confirming that specific miR-146a SNPs are associated with susceptibility to multiple sclerosis (MS) and systemic lupus erythematosus (SLE) (51). miRNA regulation can also change in response to genetic variants in the 3′ untranslated region (UTR) of mRNA targets that may affect mRNA stability, translation and miRNA binding. An SNP inside the IKAROS Family Zinc Finger 3 (IKZF3) gene is predicted to create a new recognition site for miR-326 and lead to significantly lower levels of IKZF3 in subjects carrying the allele. IKZF3 is a transcription factor important for B-cell activation, and the lack of this gene causes a lupus like syndrome in mice, suggesting a role for the regulatory loop of IKZF3 and miR-326 in autoimmunity (52). On the other hand, Steri et al. described a genetic variant located in the 3′UTR of the TNF Superfamily Member 13 (TNFSF13B) gene which shortens the untranslated region and deletes a miR-15a binding site. As a consequence, the protein encoded by this mRNA, BAFF, a soluble cytokine important for B cell development, and differentiation, increases in the blood of variant individuals, leading to augmented circulating B cells and immunoglobulins and an increased susceptibility to MS and SLE (53). A significant effort of data integration has more recently linked the prediction of SNPs affecting miRNA binding sites, statistics from 12 studies on different autoimmune diseases, public expression quantitative trait locus (eQTL) data and mRNA/small RNA-seq data and succeeded to reveal new autoimmune disease non-coding risk SNPs that might be involved in the miRNA-dependent causal mechanisms, providing valuable information for further functional studies (54).

## miRNAs as Potential Therapeutic Targets in Autoimmunity

The capability of miRNAs to skew Th subset differentiation candidates them as therapeutic targets in autoimmune conditions. T cell-specific miR-17–92 deficiency reduces Th17 differentiation and ameliorates EAE symptoms, identifying this miRNA cluster as a potential target for the clinical intervention of MS (55). miR-155 expression is found highly elevated in heart tissue in an inflammatory cardiac disease driven by autoantigen-specific CD4^+^ Th cells (experimental autoimmune myocarditis, EAM) and miR-155 inhibition results in attenuated severity of disease and cardiac injury, reduced Th17 immune response, and decreased dendritic cell function of secreting Th17-polarizing cytokines. Th cells from miR-155-inhibited EAM mice exhibit reduced proliferation and IL-17A secretion in response to autoantigens. These findings demonstrate that miR-155 adversely promotes inflammation by driving a Th17/Treg imbalance in favor of Th17 cells, and anti-miR-155 treatment can significantly reduce the autoimmune response (56). miR-155 was also proposed as a therapeutic target in a model of Th1/Th17-related inflammation during chronic cardiac rejection (57). Furthermore, *in vivo* silencing of let-7e, found up-regulated in Th cells of EAE mice, is able to inhibit encephalitogenic Th1 and Th17 cells and attenuate the disease, with reciprocal promotion of Th2 cell maturation (58). miR-340 is increased in memory Th cells from patients with MS, and favors pro-inflammatory Th1 responses while inhibiting Th2 cell development. These effects are mediated by IL-4 direct suppression, resulting in decreased GATA3 levels, and a Th2 to Th1 cytokine shift; treatment of Th cells from MS patients with miRNA inhibitors leads to the restoration of Th2 responses (59). Finally, miR-146a-deficient mice develop more severe EAE, with Th cells being more prone to differentiate into Th17 cells. In these animals, an enhancement of IL-6- and IL-21-induced Th17 differentiation pathway suggests miR-146a functions as a molecular stop signal for this autocrine pathway in autoreactive cells, and highlights miR-146a potential as a therapeutic target for treating autoimmune diseases (60).

## The Extracellular Vesicle-associated miRNAs as Novel Mediators of Inflammation

Most cells in the body release membrane bound vesicles of nanometric size (from 50 nm to 1 micron), either formed by the inward budding of multi-vesicular endosomes and subsequent fusion to the plasma membrane (exosomes), or directly budding from the plasma membrane (61, 62). Vesicle lumen contains miRNAs and other non-coding RNAs, not randomly but instead preferentially exported (63–67). Th subsets also release miRNAs not passively mirroring specific signatures at the intracellular level (68, 69). miRNA expression in Treg-cell-derived exosomes are distinct from that of pro-inflammatory Th1/Th17 subsets, suggesting a regulatory mechanism enforcing subset-specific vesicular diversity (69, 70). Extracellular vesicles (EVs) play an important role in T cell-to-cell communication, intervening in antigen presentation, cell stimulation, differentiation, cell killing, cytokine transport and stability, tolerance induction and allograft rejection (71–84). In both human and mouse, gene silencing mediated by miRNA-containing EVs was shown to participate into Treg-dependent immune suppression (69, 70).

The hypothesis that miRNA release into the microenvironment adds a further mechanism of plasticity to fine-tune specific Th responses at the paracrine level *in vivo*, is strengthened by the finding of miRNA-containing EVs in all tested biological fluids [blood, urine, saliva, breast milk, among others (85–92)], that suggests also an endocrine role. Very recently, systemic extracellular miRNA dysregulation in MS was implicated in the reduced frequency and dysfunctional suppression of Treg cells in disease. Kimura et al. showed that induction of human IFN-γ^−^IL-17A^−^FOXP3^+^CD4^+^ T cells is inhibited in the presence of patient (compared with healthy) blood exosomes, and that the exosomal miRNA profile of patients is characterized by significantly higher level of let-7i, able to target insulin like growth factor 1 receptor (IGF1R) and TGFBR1 in naïve Th cells (upon up-take of let-7i containing exosomes) and suppress induction of Treg cells, thus fueling MS pathogenesis (93).

Therefore, extracellular miRNAs may represent novel pathogenic mediators in the onset of autoimmune reactions and potential therapeutic targets in these diseases.

## Conclusions

miRNAs are “rheostats,” capable to fine-tune mammalian gene expression. Single miRNAs may only marginally regulate target genes but, when the cell responds to environmental changes, the coordinated modulation of tens of miRNAs altogether is a powerful strategy to efficiently affect many components of a genetic network. We have described the most relevant examples, but a more exhaustive list of miRNA-dependent cytokine modulation is reported in Table 1.

**Table 1:** A list of bibliographic references for the reported functional links between miRNAs and cytokines (either direct or indirect), ranked according to miRNA nomenclature.

	**Direct or Indirect Cytokine Target**	**System**	**Cellular Type**	**PMID**
miR-7	IL-6 [↑]	Human	PBMCs	27749601
miR-9	IL-2 [↑] IFN-γ [↑]	Human	CD4^+^ T cells	22585398
miR-10a	IL-12 [↓]	Human	Dendritic cells	25281418
	IL-23 [↓]		CD4^+^ T cell	
	IFN-γ [↑]	Human	Treg cells	23825948
miR-10b	IL-17A [↓]	Human	CD4^+^ T cells Th17 cells	28039186
miR-15a/16-1	IL-22 [↓]	Mouse	CD4^+^ T cells	29023933
miR-17, miR19b (miR-17~92)	IFN-γ [↑]	Mouse	CD4^+^ T cells	26138686
[-10pt]	IFN-γ [↑]	Mouse	Th1 cells	21972292
miR-18 (miR-106~363)	IL-17A [↓]	Mouse	CD4^+^ T cells	28617945
miR-19 (miR-17~92)	IL-4 [↑] IL-5 [↑] IL-13 [↑]	Human	CD4^+^ T cells	25362490
miR-20a-5p (miR-17~92)	IL-17 [↓]	Human	CD4^+^ T cells	28972028
miR-20a (miR-17~92)	IL-2 [↓] IL-6 [↓] IL-8 [↓] IL-10 [↓]	Human	CD4^+^ T cells	25884400
miR-20b	IL-17 [↓]	Mouse	CD4^+^ T cells	24842756
miR-21	IL-4 [↑] IL-5 [↑] IL-12-p35 [↓] IL-13 [↑]	Mouse	CD4^+^ T cells CD8^+^ T cells	28379062
	TGF-β [↓]	Human	Plasma Treg cells	26383248
	TGF-β [↓]	Mouse	Bone marrow MSC	26086742
	TNF-α [↑] IFN-γ [↑] IL-17A [↑]	Mouse	T cells	23395552
	IL-12 [↓] IL-4 [↑] IFN-γ [↓]	Mouse	Dendritic cells CD4^+^ T cells	21849676
miR-23a cluster	IFN-γ [↓]	Human	CD8^+^ T cells	25030422
miR-24	IFN-γ [↓]	Human	CD4^+^ T cells	24704866
miR-25 (miR-106b~25)	TGF-β [↓]	Human	Treg cells	20637509
miR-26a	IL-6 [↓]	Mouse	CD4^+^ T cells	25728641
miR-27	IL-4 [↓] IL-5 [↓]	Human, mouse	CD4^+^ T cells	22088562
miR-29	IFN-γ [↓]	Human	CD4^+^ T cells	22772450
	IL-32nonα [↓]	Human	PBMCs CD4^+^ T cells CD14^+^ monocytes	25808800
	IFN-γ [↓]	Mouse	CD4^+^ T cells, CD8^+^ T cells	21706005
	IFN-γ [↓]	Mouse	CD4^+^ T cells	21820330
miR-30a	IL-17A [↓]	Human, Mouse	CD4^+^ T cells	27581464
	IL-17A [↓] IL-17F [↓]	Human, Mouse	CD4^+^ T cells	27006279
miR-31	IFN-γ [↑] IL-2 [↓] IL-4 [↓]	Human	CD4^+^ T cells	26978146
	IL-2 [↑]	Human	T cells	23303246
miR-101	IL-2 [↓]	Human	CD4^+^ T cells	27898347
miR-106a (miR-106~363)	IL-17A [↓]	Mouse	CD4^+^ T cells	28617945
miR-106b (miR-106b~25)	TGF-β [↓]	Human	Treg cells	20637509
miR-125b	CCL4 [↓]	Human	Monocytes CD8^+^ T cells	25620312
	IFN-γ [↓] IL-2 [↓]	Human	CD4^+^ T cells	21706005
miR-126	IFN-γ [↓]	Mouse	CD4^+^ T cells	28987000
miR-128	IL-4 [↓] IL-5 [↓]	Human, mouse	CD4^+^ T cells	22088562
miR-146a	IL-6 [↓] IL-21 [↓]	Mouse	CD4^+^ T cells	28872459
	TGF-β [↑]	Mouse	Dendritic cells	26700406
	IL-10 [↑]	Mouse	Monocytes	26526003
	IFN-γ [↓] IL-2 [↓] IL-17 [↓]	Mouse	T cells	22891274
miR-150	IL-10 [↑]	Human	CD4^+^ T cells	26746193
	IL-2 [↓] TNF-α [↓]	Human	CD4^+^ T cells	26549736
miR-155	IL-17 [↑]	Human	CD4^+^ T cells	28471953
	IL-6 [↑] IL-23 [↑] IL-1β [↑] TNF-α [↑] IL-17A [↑]	Mouse	Dendritic cells CD4^+^ T cells	27052830
	IFN-γ [↑] IL-17 [↑]	Rat	CD4^+^ T cells	26349986
	IL-21 [↑]	Human	CD4^+^ T cells	26055806
	IL-17 [↑]	Human	CD4^+^ T cells	25761610
	IL-17 [↑]	Mouse	Dendritic cells CD4^+^ T cells	25651871
	IFN-γ [↑]	Mouse	CD4^+^ T cells	24891206
	IL-13 [↑]	Mouse	CD4^+^ T cells	25024218
	IL-9 [↑] IL-10 [↑] IL-22 [↑]	Mouse	CD4^+^ T cells	24856900
	IL-2 [↓]	Human	CD4^+^ T cells	22785227
	IL-17 [↑]	Mouse	Th17 cells	23686497
	IL-17 [↑]	Mouse	Th17 cells	23091595
	IFN-γ [↑]	Mouse	T cells	23200854
	IL-17A [↑] IL-6 [↑] IL-12 [↑] IL-23 [↑] TNF-α [↑]	Mouse	CD4^+^ T cells Dendritic cells	20888269
	IL-4 [↓] IL-5 [↓] IL-10 [↑]	Mouse	CD4^+^ T cells	17463290
miR-181	IFN-γ [↓]	Human	CD4^+^ T cells	24704866
miR-181c	IL-2 [↓]	Human	CD4^+^ T cells	21112091
miR-182	IL-2 [↓]	Human	Treg cells	23825948
miR-200a	IL-17 [↑] IL-23 [↑]	Human	CD4^+^ T cells	28738533
	IL-2 [↑]	Mouse	CD4^+^ T cells	28438897
miR-210	TNF-α [↑]	Human	CD8^+^ T cells	27749601
	IL-17 [↓]	Mouse	T cells	24608041
miR-212/132	IL-10 [↓]	Mouse	CD4^+^ T cells	25862525
	IL-17 [↑]	Mouse	CD4^+^ T cells	23818645
miR-301a	TNF-α [↑] IL-17 [↑]	Human Mouse	CD4^+^ T cells Th17 cells	26338824
	IL-17 [↑]	Mouse	CD4^+^ T cells	22517757
miR-326	IL-17 [↓]	Human	CD4^+^ T cells	27454344
	IL-17 [↑]	Human	Th17 cells	19838199
miR-340	IL-4 [↓] IL-5 [↓]	Human, mouse	CD4^+^ T cells	22088562
*miR-363-3p* (miR-106~363)	IL-17A [↓]	Mouse	CD4^+^ T cells	28617945
miR-425	IL-2 [↓] IFN-γ [↓]	Human	CD4^+^ T cells	28192189
Let-7 family	IL-10 [↓]	Human	CD4^+^ T cells	22586040
	IL-13 [↓]	Human	T cells	21616524
Let-7a	IL-13 [↓]	Mouse	CD4^+^ T cells	20630862
Let-7e	IL-4 [↓] IL-10 [↓] IL-17 [↑] IFN-γ [↑]	Mouse	CD4^+^ T cells, CNS-mononuclear cells	23079871
Let-7f	IL-17 [↓]	Human	CD4^+^ memory T cells	21508257
Let-7i	IL-2 [↑]	Human	CD4^+^ T cells	27145859
	IL-10 [↓]	Rat	Dendritic cells	26755202

*The species in which the observation was made and the cell type are also registered*.

Studies in different Th subsets concur to show that miRNAs are able to direct differentiation by restraining the expression of genes “*inappropriate*” to that specific cell subset, including cytokines characterizing the function of other subsets. Furthermore, master regulatory transcription factors positively induce Th differentiation also through “repression of miRNA-based repression” of genes “*appropriate*” to that specific cell subset. In most cases, a single miRNA targets different sets of mRNAs depending on cell context and the co-expression of other miRNAs and/or higher affinity gene targets, resulting in different functional outputs. Finally, the contiguity of different Th subsets, or better their (not yet completely revealed) plasticity, is also evident when considering that the same miRNAs are crucial in the differentiation of functionally divergent subsets such as Th1/17 and Treg. Hence, we need to not only identify *which* miRNAs regulate *which* cytokines but also frame the mechanistic miRNA regulation in a *subset-specific context*. The picture is further complicated by EV-associated miRNAs traveling in the extracellular space and becoming regulatory signals in cell-to-cell communication likewise cytokines themselves.

In conclusion, if we want to take advantage of the powerful regulatory action of miRNAs for therapeutic purposes, in the next years we will have to fully untangle the intricate web of miRNA-target genes to safely re-direct the differentiation and function of CD4^+^ Th cell subsets in pathological conditions such as autoimmunity.

## Author Contributions

PdC conceived the article. PdC and SG collected and assembled data and wrote the manuscript. VD substantially contributed to draft writing and critical revision of the article. All authors approved the final version of the manuscript.

### Conflict of Interest Statement

The authors declare that the research was conducted in the absence of any commercial or financial relationships that could be construed as a potential conflict of interest.
